# Metronome-guided cochlear implantation for slower and smoother insertions of lateral wall electrodes

**DOI:** 10.1007/s00405-024-08639-4

**Published:** 2024-04-17

**Authors:** W. Wimmer, Julia Veloso de Oliveira, T. M. Breitsprecher, S. Hans, V. Van Rompaey, P. Van de Heyning, S. Dazert, Nora M. Weiss

**Affiliations:** 1 Department of Otorhinolaryngology, Klinikum Rechts Der Isar, TUM School of Medicine and Health, Technical University of Munich, Munich, Germany; 2 Department of Otorhinolaryngology-Head and Neck Surgery, Ruhr-University Bochum, St. Elisabeth-Hospital Bochum, Bochum, Germany; 3 MED-EL, Elektromedizinische Geräte, Innsbruck, Austria; 4 Department of Translational Neurosciences, Faculty of Medicine and Health Sciences, University of Antwerp, Antwerp, Belgium; 5 Department of Otorhinolaryngology and Head & Neck Surgery, Antwerp University Hospital, Antwerp, Belgium; 6 International Graduate School of Neuroscience (IGSN), Ruhr-University Bochum, Bochum, Germany

**Keywords:** Hearing preservation, Insertion monitoring, Robotic cochlear implantation, Insertion friction, Free fitting electrode array

## Abstract

**Introduction:**

Achieving a slow and smooth electrode array insertion is paramount for preserving structural and functional integrity during cochlear implantation. This controlled study evaluates the efficacy of a metronome-guided insertion technique in enhancing the smoothness and speed of electrode array insertions.

**Methods:**

In a prospective cohort study, patients undergoing cochlear implant surgery between 2022 and 2023 with lateral wall electrode arrays were included. Metronome guidance was delivered through an acoustic signal via headphones during electrode array insertion in cochlear implantation and compared to a control group without metronome-guidance.

**Results:**

In total, 37 cases were evaluated, including 25 conventional insertions and 12 metronome-guided insertions. The results indicate that metronome-guided insertions were significantly slower (− 0.46 mm/s; *p* < 0.001) without extending the overall procedure time. This can be attributed to fewer paused sections observed in the metronome-guided technique. Moreover, metronome-guided insertions exhibited superior performance in terms of insertion smoothness and a reduced number of re-gripping events.

**Conclusions:**

The findings support the recommendation for the systematic application of metronome guidance in the manual insertion of cochlear implant electrode arrays, emphasizing its potential to optimize surgical outcomes.

**Supplementary Information:**

The online version contains supplementary material available at 10.1007/s00405-024-08639-4.

## Introduction

Cochlear implantation is the therapy of choice to treat severe to profound sensorineural hearing loss. The indication for cochlear implantation also includes cases with residual hearing increasing the need for structure-preserving surgical techniques.

An important factor shown to influence the outcome of cochlear implantation is the insertion speed of the electrode array. For lateral wall electrode arrays, a slow insertion speed leads to higher rates of complete array insertions, reduces the occurrence of intracochlear resistance, and assists the preservation of residual hearing and vestibular function [1]. In contrast, faster speeds significantly increase insertion forces [2–4] and are associated with intracochlear array translocations [5]. These findings may explain the negative impact of faster array insertions on residual hearing [6]. In this context, a constant and slow insertion with a speed of 0.25 mm/s is considered advantageous [7]. Nevertheless, it is argued that insertion speeds of 0.25 mm/s are not achievable by humans who are haptically limited to speeds above 0.87 mm/s [8].

In addition to speed, unsteadiness of the electrode array movement during insertion such as re-gripping may lead to intracochlear pressure peaks and thus to an increased risk of trauma [9–11]. Automated insertion techniques have been introduced [7, 12, 13] and their effectiveness has been shown and is currently further clinically evaluated [14–16]. However, these require additional equipment, training and installation effort and impose additional costs. Furthermore, investigations toward improved insertion dynamics during manual procedures would be generally valuable for clinical routine.

People's sense of time and duration is flexible and can be distorted by speed, as highlighted in research on time perception in human movement [17]. It is widely accepted that surgical skills may be trained [18] and that surgeons can improve their skills by combining visual cues, awareness of body position (proprioception), and feedback from touch [19]. Nevertheless, the amount in which a sense of speed may reliably be estimated by oneself and by which other factors it is influenced is not clear [17]. While humans may not match the precision of specialized robotic tools, training and guidance can significantly enhance the performance of manual tasks. Training is achieved by performing a sufficient number of surgical interventions. In addition, feedback could improve the surgical technique [20, 21]. In cochlear implantation where the insertion speed is known to influence the surgical outcome, one way to achieve a slower and smoother array advancement would be to provide an additional “clock” reference signal (i.e., a “tact” or “metronome”). For example, there is evidence that metronome guidance can help to improve the adequacy of chest compression [22]. In addition, it was demonstrated that the sense of metronomic speed estimation can be trained [23]

In this study, we present the application of metronome guidance in cochlear implantation aiming at improving the manually performed electrode array insertion technique. By presenting an acoustic metronome signal via headphones, we hypothesized that slower insertion speeds and overall smoother array insertions could be achieved.

## Methods

### Study design and participants

To test the hypothesis, two insertion techniques, i.e., “conventional” vs. “metronome-guided” were prospectively compared in clinical cases. In total, 37 patients (25 women, 12 men, 15 left ears, 22 right ears) receiving a cochlear implant between June 2022 and May 2023 were included in the study. The mean age of the patients was 54 years (SD 19 years). Four of the cases were revision cases.

### Ethical statement

The study protocol was approved by the local institutional review board in accordance with the Helsinki declaration (Reg.-No.: 21-7373). Written informed consent was obtained from all participants.

### Surgical approach and insertion technique

Two expert otologists performed the implantations. For all cases, audio (surgeons’ own comments) and video recordings (microscopic view, 25 frames per second) were taken during the surgical procedure for later analysis using an open source software (OBS Studio) [24]. To prepare electrode array insertion, a retroauricular approach including a mastoidectomy and facial recess approach was performed in all cases. Thirteen patients were implanted with Flex^28^ arrays, 17 received Standard arrays, and 7 Flex Soft arrays [all arrays with 12 electrode contacts (contact 1: most apical, contact 12: most basal), MED-EL, Innsbruck, Austria]. All arrays were inserted through the round window.

**Fig. 1:**
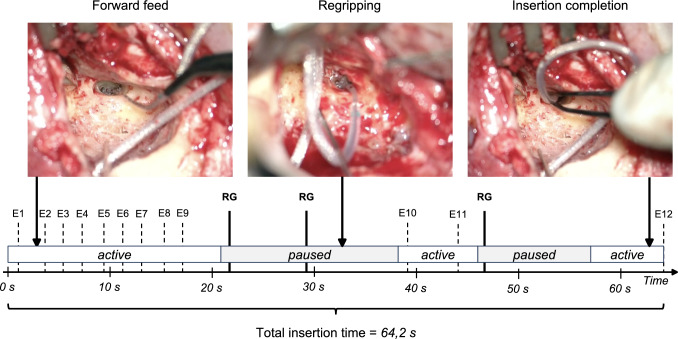
Timeline with video recordings during electrode array insertion using the conventional technique (case 23). Active and paused insertion phases, electrode insertion events (E), and re-gripping events (RG) as well as the total insertion time are indicated

Two different insertion techniques were used by the surgeons. First, in 25 of 37 cases, a “conventional” technique without receiving any instructions or feedback during the insertion was performed. The remaining 12 cases were “metronome-guided”, i.e., an acoustic metronome signal was presented via headphones as additional feedback for reference (Fig. 1). For this purpose, a 500 Hz sine pulse was started with the beginning of the insertion and presented every 10 s using an open source audio editor (Audacity®). The surgeons were instructed to aim for inserting one electrode contact per acoustic pulse.

### Data analysis and outcome measures

To quantify the speed and consistency of the insertions, the recorded audio and video material were assessed for several outcome measures. For each inserted electrode contact, time markers (40 ms resolution) were identified by two observers (NMW and WW) based on the microscopic view and surgeon comments. Annotations were made under common consensus of both observers. We categorized two phases during array insertion, depending on whether the array was effectively advanced (“active insertion”) or not (“paused insertion”, e.g., open forceps) (see Fig. 1).

#### Total insertion time (in s)

The total insertion time was defined as the duration between start of the insertion (first electrode at the round window) until full insertion (twelfth electrode within the round window). The first electrode contact was used as reference, because the silicone tip of the arrays was not reproducibly determinable in the material.

#### Effective insertion time (in s)

In contrast to the total insertion time, which included paused insertion phases, the effective insertion time was defined as the sum of all active insertion phases during an insertion.

#### Contact insertion time (in s)

In addition, for each electrode contact, the total duration taken for insertion of an electrode contact was calculated.

#### Averaged insertion speed (in mm/s)

Subsequently, the averaged insertion speed was computed by dividing the length between the first and the twelfth electrode contact (23.1 mm for Flex^28^ arrays, 26.4 mm for Flex^Soft^ and standard arrays) by the effective insertion time.

#### Contact insertion speed (in mm/s)

The active insertion time taken to insert an electrode contact divided by distance between two contacts (2.1 mm for Flex^28^ arrays, 2.4 mm for Flex^Soft^ and standard arrays).

#### Insertion smoothness (in mm/s)

The continuity of the insertion speed was evaluated by calculating the standard deviation of the individual contact insertion speed among a case.

#### Number of re-gripping events

As additional outcome measure for continuity, array re-grippings were counted, i.e., how often the forceps were opened to (re-)grab the array during insertion.

### Statistical analysis

Two-sided Wilcoxon rank-sum tests were applied to assess the statistical significance of the observed differences between the insertion techniques for the total and effective insertion time, the averaged insertion speed, the insertion smoothness, and the number of re-gripping events.

For the statistical analysis of contact insertion time and contact insertion speed, two distinct linear mixed-effects models were fit. In both models, the insertion technique (‘conventional’ vs. ‘metronome-guided’) and its interaction with the electrode contact number (treated as a continuous standardized variable) were included as fixed effect, to specifically assess whether contacts exhibit differences in insertion time and speed. In addition, the array type (‘Standard,’ ‘Flex^Soft^’, or ‘Flex^28^’) was included as control variable. To accommodate the repeated measures inherent in the data, subjects were included as random intercepts. The models were fitted using the lme4 package [25]. The threshold for statistical significance was set to *α* = 0.05. Statistical analysis was performed using the R Studio environment [26]. Data visualization was performed using the Python ‘seaborn’ library [27].

## Results

### Array insertion time

**Fig. 2:**
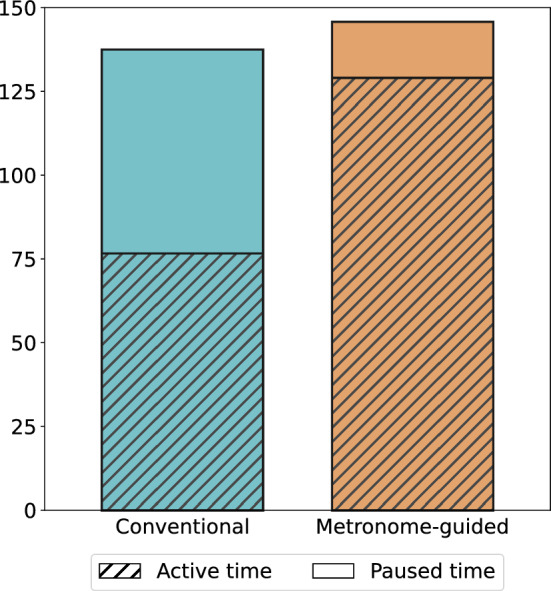
Averaged total, effective and paused insertion times for the conventional and the metronome-guided insertion cases. While the total insertion time is comparable between the two techniques, the proportion of time with an effective array advancement into the cochlea (effective insertion time) was significantly lower for the conventional (56% of the total time) than the metronome-guided technique (88% of the insertion time)

We found no statistically significant difference (*p* = 0.15) for the total insertion time between the conventional and metronome-guided insertion cases, with an average of 137 s (SD 117 s) and 146 s (SD 39 s), respectively (Fig. 2). However, a noticeable difference between the techniques was observed for the averaged effective insertion time, accounting for 77 s (SD 51 s) in the conventional technique compared to 129 s (SD 18 s) in the metronome-guided technique (*p* < 0.001). This corresponds to a proportion of effective to total insertion time of 56% and 88% for the conventional and metronome-guided technique, respectively.

### Contact insertion time

The conventional technique had a shorter average and median contact insertion time (11.2 s and 3.6 s, respectively) compared to the metronomic insertion technique (13.0 s and 10.5 s, respectively). The linear mixed-effects model showed no statistically significant difference in contact insertion between the insertion techniques (2.5 s; *p* = 0.45). Moreover, there was no statistically significant difference observed in contact insertion times among individual electrode contacts when using metronome-guidance. In contrast, using the conventional insertion technique, higher electrode contact numbers were correlated with longer insertion times (*p* < 0.001). No variations were observed among electrode array types. For an overview of the fit model, please refer to Table S1 in the supplementary material.

### Averaged insertion speed

**Fig. 3:**
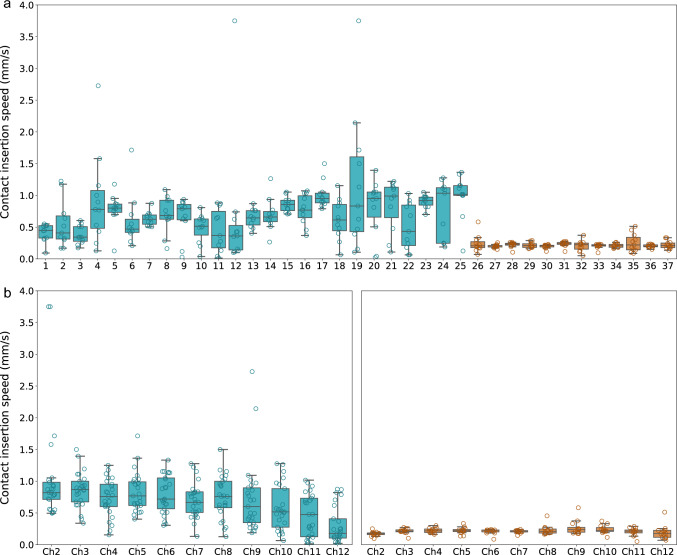
Contact insertion speed summarized in boxplots for mean of individual cases (**a**) and mean individual electrode contacts (**b**). Blue and orange bars indicate conventional and metronome-guided insertions, respectively. Bars indicate interquartile ranges. Lines indicate medians. Whiskers indicate minimum and maximum value distribution with exception of outliers

There was a statistically significant difference (*p* < 0.001) of the average insertion speed between the metronome-guided (0.19 mm/s; SD 0.03 mm/s) and the conventional technique (0.45 mm/s; SD 0.22 mm/s; Fig. 3).

### Contact insertion speed

Figure 3 summarizes the insertion speeds for individual cases and techniques, highlighting the impact of insertion techniques on contact insertion speeds. The mean and median contact insertion speeds were higher for the conventional technique (0.71 mm/s and 0.70 mm/s) compared to the metronome-guided technique (0.22 mm/s and 0.21 mm/s). The linear mixed-effects model revealed a significant reduction in speed by 0.46 mm/s (*p* < 0.001) for metronome-guided insertions. Moreover, within the metronome-guided cases, no variation in insertion speed was observed among individual electrode contacts. Conversely, with the conventional insertion technique, an increase in contact number (i.e., contacts inserted toward the end) was associated with a decreasing insertion speed (*p* < 0.001). Notably, electrode array types did not show a statistically significant effect on contact insertion speed. Refer to Table S2 in the supplementary material for an overview of the fit model.

### Insertion smoothness

The averaged standard deviation of the contact insertion speed per case was statistically significantly lower (*p* < 0.001) with the metronome-guided technique (0.06 mm/s, SD 0.04 mm/s) compared to the conventional technique (0.35 mm/s, SD 0.25 mm/s). This indicates overall smoother insertions under metronome guidance.

### Number of re-grippings

The metronome-guided technique led to a significantly lower averaged number of re-gripping events (3.0, SD 3.7) compared to the conventional technique (5.6, SD 4.2*; p* = 0.033).

## Conclusion

We found that metronome guidance leads to overall slower, steadier and smoother electrode array insertions and fewer re-gripping events. Even though comparable surgical times for the insertion of the electrode array were achieved, these were more efficiently used in the metronome-guided cases.

The speeds of insertion found in our study align with the findings of Rajan et al. who reported insertion speeds ranging from 0.25 to 1.00 mm/s [1]. Another study performed by Kontorinis et al. reported significantly higher insertion speeds with an average of 1.6 mm/s [2]. However, this group observed significant variances in the insertion speed depending on the surgeon. Another study investigating insertion speed in a model report that slow insertions are feasible, but the speed limit for constant movements was reported to be 0.87 mm/s [8]. Thus, Kesler et al. hypothesize that a limit below 0.87 mm/s is not achievable for a continuous forward insertion in manual cochlear implantation. However, the study concludes that the results are limited by the sample size and that larger sample sizes need to be investigated to draw conclusions concerning the level of training and insertion abilities. In our study, metronome-guided insertion speeds were substantially slower than these reported values. Nevertheless, we were able to demonstrate that the standard deviation of the contact insertion speed per case was significantly lower in the metronome-guided group indicating that metronome guidance enables significantly slower but still constant insertion speeds. Furthermore, the observed average 0.22 mm/s contact insertion speed in the metronome-guided cases are corresponding well to the theoretical insertion speed of about 0.23 mm/s (Flex28) and 0.26 mm/s (FlexSoft and Standard). The feasibility of insertion speeds below 0.87 mm/s was also demonstrated in cochlear models [9].

We observed slower insertion speeds of the basal electrode contacts in the control group compared to the metronome-guided group. This is in line with another study from Aebischer et al. in which this trend was observed. Consequently, they argued that particularly in the last phase of the electrode array insertion increasing forces imply a large part of the total insertion energy being applied in a short period of time and may thus increase the cochlear damage [9]. Increased insertion forces at the end of implantation may lead to electrode array kinking and thus to intracochlear trauma such as fractures of the osseous lamina [28, 29]. In the metronome-guided group, this phenomenon could not be observed leading to the assumption, that the simple application of a metronome guidance leads to a steadier distribution of the insertion forces and may improve the surgical outcome. This is also underlined by the finding, that re-gripping events were significantly lower in the metronome-guided group indicating, that the insertion process is more effort and frictionless compared to the control group.

As a side note, we found that the surgeon experienced the insertion as more friction-free in the metronome-guided cases. This is in line with the findings from Rajan et al. that found a higher rate of complete insertions in cases with slow insertion speeds [1]. One possible explanation is that the slow speed allows the flexible electrode array to unfold in the perilymph. Another study from Aebischer et al. strengthens this hypothesis by showing that peak forces can be reduced with alignment angles parallel to the scala tympani [3]. Based on our results, it may be assumed, that metronome-guidance offers some of the advantages of robot-assisted insertion techniques while it is cheaper and broader available.

This study is limited by a small number of cases in the metronome-guided group. However, we consider the strong effects that we observed significant enough to draw conclusions with a severe surgical impact. Furthermore, we are aware, that manual insertion is not able to be reliably continuous. Nevertheless, we were able to demonstrate that the metronome reduced discontinuities in a large extend. Furthermore, this study does not discuss the technique’s influence on speech perception outcomes. Demonstrating improved functional outcomes necessitates a significantly larger sample size, especially given the considerable variability in outcomes among cochlear implant recipients and the multitude of confounding variables influencing postoperative speech understanding. The absence of randomization in our study design could limit the ability to discern a genuine effect across different cohorts. However, we consider the technique valuable, since it is easily implemented in clinical routine, regardless of the setting, e.g., the availability of robotic tools.

To conclude, the use of metronome guidance offers three major improvements in cochlear implant surgery. First, we demonstrated, that slower insertion speeds are achieved, second, that the insertion is more constant and third fewer re-grippings are needed. All, slow and smooth insertion as well as reduced movements have been proven to be advantageous for hearing and structure preservation. Consequently, we recommend the broad application of metronome guidance during the insertion of a cochlear implant electrode array analogous to the one in chest compression.

## Supplementary Information

Below is the link to the electronic supplementary material.

Supplementary file1 (DOCX 14 KB)

Supplementary file2 (DOCX 13 KB)

## Data Availability

Data is available on special request.
