# Supplementary material for: Metronome-guided cochlear implantation for slower and smoother insertions of lateral wall electrodes
Source: Eur Arch Otorhinolaryngol. 2024 Apr 17;281(9):4603–9. doi: 10.1007/s00405-024-08639-4 (PMC11393022; doi:10.1007/s00405-024-08639-4)
Supplement: Supplementary file 1 — Supplementary file1 (DOCX 14 KB) [file 405_2024_8639_MOESM1_ESM.docx]

**Supplemental Tables**

**Table S1.** Linear mixed-effects model results for contact insertion time.

Linear mixed model fit by REML. t-tests use Satterthwaite's method ['lmerModLmerTest']

Formula: TIME_PER_ELECTRODE ~ METHOD + METHOD:ELECTRODE + TYPE + (1 | ID)

Data: data

REML criterion at convergence: 3616.8

Scaled residuals:

Min 1Q Median 3Q Max

-1.3095 -0.3942 -0.1103 0.1302 9.4862

Random effects:

Groups Name Variance Std.Dev.

ID (Intercept) 39.04 6.248

Residual 424.33 20.599

Number of obs: 407, groups: ID, 37

Fixed effects:

Estimate Std. Error df t value Pr(>|t|)

(Intercept) 11.4216 3.0415 33.0000 3.755 0.00067 ***

METHODMETRONOMIC 2.5337 3.3365 33.0000 0.759 0.45301

TYPEFLEXSOFT -3.2507 3.8892 33.0000 -0.836 0.40927

TYPESTANDARD 0.8135 3.5895 33.0000 0.227 0.82211

METHODCONVENTIONAL:ELECTRODE 10.0515 1.2437 368.0000 8.082 9.27e-15 ***

METHODMETRONOMIC:ELECTRODE 1.6167 1.7951 368.0000 0.901 0.36840

---

Signif. codes: 0 ‘***’ 0.001 ‘**’ 0.01 ‘*’ 0.05 ‘.’ 0.1 ‘ ’ 1
