# Supplementary material for: Metronome-guided cochlear implantation for slower and smoother insertions of lateral wall electrodes
Source: Eur Arch Otorhinolaryngol. 2024 Apr 17;281(9):4603–9. doi: 10.1007/s00405-024-08639-4 (PMC11393022; doi:10.1007/s00405-024-08639-4)
Supplement: Supplementary file 2 — Supplementary file2 (DOCX 13 KB) [file 405_2024_8639_MOESM2_ESM.docx]

**Supplemental Tables**

**Table S2.** Linear mixed-effects model results for contact insertion speed.

Linear mixed model fit by REML. t-tests use Satterthwaite's method ['lmerModLmerTest']

Formula: SPEED ~ METHOD + METHOD:ELECTRODE + TYPE + (1 | ID)

Data: data

REML criterion at convergence: 300.7

Scaled residuals:

Min 1Q Median 3Q Max

-3.1603 -0.4112 -0.0316 0.2009 8.5492

Random effects:

Groups Name Variance Std.Dev.

ID (Intercept) 0.01695 0.1302

Residual 0.10591 0.3254

Number of obs: 407, groups: ID, 37

Fixed effects:

Estimate Std. Error df t value Pr(>|t|)

(Intercept) 0.646527 0.056286 33.000000 11.486 4.51e-13 ***

METHODMETRONOMIC -0.458134 0.061744 33.000000 -7.420 1.59e-08 ***

TYPEFLEXSOFT 0.031159 0.071973 33.000000 0.433 0.668

TYPESTANDARD 0.106209 0.066426 33.000000 1.599 0.119

METHODCONVENTIONAL:ELECTRODE -0.176839 0.019649 368.000000 -9.000 < 2e-16 ***

METHODMETRONOMIC:ELECTRODE 0.006861 0.028361 368.000000 0.242 0.809

---

Signif. codes: 0 ‘***’ 0.001 ‘**’ 0.01 ‘*’ 0.05 ‘.’ 0.1 ‘ ’ 1
